# Prevalence and Related Factors of Lower Urinary Tract Infection in Frail Older Adults Undergoing Major Noncardiac Surgery

**DOI:** 10.3390/geriatrics8020033

**Published:** 2023-02-28

**Authors:** Warin Thangrom, Inthira Roopsawang, Suparb Aree-Ue

**Affiliations:** Ramathibodi School of Nursing, Faculty of Medicine Ramathibodi Hospital, Mahidol University, Bangkok 10400, Thailand

**Keywords:** blood glucose level, frailty, urinary tract infection, preoperative assessment, noncardiac surgery, older adults

## Abstract

Urinary tract infections are the most common complication after surgery in older adults, resulting in poor operative outcomes and reduced quality of life after discharge. However, there is limited research investigating the relationships between urinary tract infection and associated factors in frail older surgical patients, particularly in Thailand. This retrospective study included 220 frail older patients aged ≥ 60 years who had undergone major noncardiac surgery at a tertiary care hospital in Thailand from January 2015 to December 2019. The sample was recruited using the criteria indicated in the modified Frailty Index-11 and having the blood glucose level determined within 2 h before surgery. The prevalence of lower urinary tract infections was 15% post-surgery. Firth’s logistic regression analysis revealed that the equation could predict the accuracy of lower urinary tract infections by 88.5%. Frailty, blood glucose levels, complication during admission, and personal factors together predicted the variability of lower urinary tract infections. Adjusting for other variables, being an older adult with severe frailty and complications during hospital admission significantly increased the risk of developing lower urinary tract infections (odds ratio = 3.46, *p* < 0.05; odds ratio = 9.53, *p* < 0.001, respectively).

## 1. Introduction

Global aging is a phenomenon resulting in an increased number of older adults requiring surgery to regain their function and enhance their quality of life. To date, the number of noncardiac surgical procedures has increased to treat declining conditions due to aging, including degenerative joint diseases, vascular diseases, gastrointestinal disorders, and malignancies; however, postoperative complications impacting the quality of life have been extensively documented [1,2,3]. Current evidence has revealed that approximately 300 million noncardiac procedures are performed annually worldwide [4]. Older patients undergoing noncardiac surgery are more likely to experience adverse cardiovascular events or myocardial injury/infarction [5]. Clearly, the risk of developing postoperative complications increases with advanced age. Moreover, atypical symptoms or asymptomatic urinary tract infections (UTIs) are common in older adults, leading to the development of complicated UTIs that are difficult to correctly diagnose, resulting in them being frequently missed, thereby potentially increasing the risk of developing serious infections [6]. Therefore, providing efficient care for promoting health and avoiding preventable complications is more challenging in the older surgical population.

UTIs can cause minor complications in other populations but have significantly more severe consequences for older adults, particularly those with chronic health conditions. Recent evidence has demonstrated that UTIs, particularly those of the lower tract, are the most prevalent hospital infections, accounting for 25% of hospitalizations, which not only leads to high care costs but also increases detrimental events in older hospitalizations [7]. Furthermore, following surgery, UTIs are a significant cause of developing sepsis, greatly increasing healthcare costs, and causing up to 40% of in-hospital mortality [8,9,10]. In addition, recent evidence has proposed that the age-related decline condition known as frailty plays an essential role in increasing functional deterioration with a decreased response to stressors, making a person vulnerable to poor outcomes [2,11,12,13,14,15]. Frailty is a well-established measure of surgical outcomes, leading to increased risks for developing complicated UTIs, particularly in older adults with chronic illnesses, including diabetes mellitus [16,17,18]. Because of the effects of physiological changes due to aging processes and frailty, an immune system disturbance evolves, resulting in a poor response to foreign bodies and pathogens. Diminished phagocytosis by T-cells reduces their capacity to destroy pathogens, leading to ineffective infection control, thereby increasing the risk of older adults developing infections [16,17,19]. Concurrently, frailty may interfere with glucose homeostasis, leading to pancreatic impairment—beta-cell dysfunction and increased alpha cell and glucagon secretion—resulting in dysglycemia (higher or lower blood glucose levels) [20,21]. Reduced glucose metabolism is a significant issue in the aging process, known as age-related glucose dysregulation [20,22,23]. Glucose tolerance progressively declines with age, contributing to the high prevalence of impaired glucose tolerance and Type 2 diabetes among older adults [18,23,24]. Under normal circumstances, the human regulatory systems respond to surgical stress by inducing specific mechanisms that aim to regulate the homeostasis condition. Remarkably, recent evidence has underlined that surgery typically induces altered systemic glucose metabolism [25,26]. No matter which part of the body undergoes surgery, the injury or trauma induces disorder mechanisms in glucose metabolism and innate immune responses; however, these responses can become altered in older adults [25,26,27]. In doing so, during illness, complex interactions between counterregulatory hormones and cytokines lead to an increase in excessive glucose production, which is also associated with insulin resistance [28,29]. Therefore, hyperglycemia is frequently reported in individuals with acute medical or surgical situations associated with the increased circulation of counterregulatory hormones, resulting in metabolic and hormonal alterations [20,22,23]. Several products of oxidative stress, free fatty acids, and inflammatory mediators are increased during those situations. These pathophysiologic alterations contribute to direct cellular damage, vascular malfunction, and immunological dysfunction [20,23,29,30]. Therefore, in surgical settings, older adults with or without Type 2 diabetes are at risk of experiencing postoperative complications. Identifying such risks and thereby preventing complications is essential to promote health and quality of care in this population.

Prior studies have noted that both frailty and uncontrolled glucose levels increase the risk of detrimental health outcomes in older adults undergoing surgical procedures, particularly in developing lower UTIs and adverse outcomes, a prolonged length of hospital stay, and experiencing postoperative complications or in-hospital mortality, leading to increased care costs [11,13,16,18]. Effective identification or risk stratification is essential for gerontological healthcare professionals to prevent UTIs. However, much uncertainty remains regarding the relationship between age-related decline in conditions and surgical outcomes in the noncardiac surgical population. Furthermore, the nexus linkage between frailty, diabetes, and lower UTIs was not fully explained in the context of tertiary care hospitals, where older patients with complex health conditions are more likely to have elective surgery than in primary care hospitals. As such, this study aimed to investigate the prevalence of postoperative lower UTIs and the associated factors between age, gender, diabetes mellitus (DM), preoperative levels of frailty, blood glucose levels, complications during hospital admission, and lower UTIs in older noncardiac surgical adults. The findings of the present study may not only provide a better understanding of the impact of age-related decline on postoperative complications but could also improve awareness regarding screening for frailty and blood glucose control as associated factors for lower UTIs. In addition, the modification of related factors might decrease the risk of developing lower UTIs, promote efficient care, and enhance the quality of life during treatment and after discharge in the older population.

## 2. Materials and Methods

### 2.1. Study Design

The methodological approach taken in this present study was a retrospective study design. The data were extracted from the electronic medical records of in-patient older patients aged ≥60 years scheduled for major noncardiac surgery at a tertiary care hospital in Thailand during 1 January 2015–31 December 2019.

### 2.2. Participants

The eligible participants were recruited based on randomly selected electronic medical records for independent review; the specific requirements targeting variables of interest were delineated for the review. The inclusion criteria focused on the individuals who presented with a frailty status evaluated using the modified Frailty Index-11 (mFI-11) [15] and had their blood glucose level determined within 2 h before surgery. The exclusion criteria were: (1) mortality during hospitalization, (2) referral to a different hospital for further treatment, (3) receipt of any palliative care or terminal treatment during hospital admission, and (4) incomplete medical records or missing clinical diagnoses for hospital discharge.

The sample size calculation was based on that of the logistic analysis concept by Bujang et al. [31]. The calculation formula was n = 100 + 50 (i), where “i” refers to the number of independent variables of the study: levels of frailty and blood glucose levels. Therefore, the appropriate sample size was expected to be a minimum of 200. To improve the predictive analysis, 10% of the cases were added; thus, the total number of appropriate cases was 220.

### 2.3. Tools

#### 2.3.1. Modified Frailty Index-11

The mFI-11 is an assessment tool to evaluate frailty status that originated from combining 11 clinically relevant parameters to indicate frailty [15]. The score is calculated by dividing the number of variables by the total number of variables assessed (n/11). In the study, the mFI-11 scores are classified into three categories of frailty status: pre-frail or low level of frailty (scores ≥ 0.09), frailty (scores ≥ 0.27), and severe frailty (scores ≥ 0.45). The mFI-11 is an efficient technique for risk-staging surgical patients and has been correlated with all surgical specialties. It has demonstrated good predictability in terms of adverse health outcomes, including postoperative complications, readmission, reoperation, discharge to skilled care, more extended hospital stays, and a high mortality rate [13,15]. Owner permission was granted to allow the mFI-11 to be used in the present study.

#### 2.3.2. Electronic Health Records (EMRs) Criteria for Hospital-Diagnosed Lower UTIs

In the present study, evidence of developing lower UTIs during hospitalization is considered to be based on clinical diagnoses made by a physician using standard coding for diagnosing UTIs (International Classification of Diseases (ICD)-9 or ICD-10), together with any treatments associated with UTIs and positive urinalysis and/or urine culture extracted from completed medical records. In contrast, the absence of such evidence is considered to be evidence of the absence of lower UTIs.

### 2.4. Statistical Analysis

Descriptive statistics were applied for demographic variables: gender, age, marital status, education, comorbidities, body mass index, activities of daily living, pre-operative diagnosis and operating procedure, blood glucose levels (before surgery), pre-operative urine examination, postoperative urine examination, length of stay, and postoperative complications, which were expressed as frequency, percentage, mean, and standard deviation (SD). The prevalence rate of lower UTIs is expressed as a percentage based on a five-year simple random sample. The factors associated with lower UTIs were assessed using the multiple logistic regression analysis model, controlling for age, gender, DM, and complications during hospital admission. The Firth logistic regression approach was used to eliminate bias and enhance prediction precision for rare events. The significance set at 5% (*p*-value < 0.05), the odds ratio (OR), and the 95% confidence interval were used to determine the strength of the association. Data analysis was performed using the licensed Social Sciences for Windows (SPSS) version 18 and RStudio version (4.2.2 for MAC).

### 2.5. Ethical Considerations

This retrospective study was conducted after obtaining the approval of the Human Research Ethics Committee and Institutional Review Board (number ID: MURA2021/483). All procedures were performed under the Declaration of Helsinki-Ethical principle for human data and research.

## 3. Results

### 3.1. Participant Characteristics

The participants included 220 older adults undergoing noncardiac surgery. Of these, the majority were female (62.3%) with a mean age of 72.95 years (SD = 72.53; range 60–94 years), married (72.7%), and had completed primary education (65.5%). Regarding health information, most of the participants were independent (90%), with 49.5% being overweight or obese and 71.9% having multiple comorbidities. At the preoperative stage, 28.6% had high blood glucose levels, and approximately half of the participants were frail or severely frail (53.6%). Overall, the length of the hospital stay was 11.9 days (ranging from 2 to 132 days, SD = 14.7). For surgical treatments, gastrointestinal surgery was the most prevalent among the noncardiac surgical procedures (40.9%). Postoperative complications occurred in 20.9% of patients, of whom approximately 20% developed lower UTIs post-surgery. The details are presented in Table 1.

Considering lower UTIs and frailty status, the more severe the frailty status, a higher rate of postoperative lower UTIs was identified after undergoing major noncardiac surgery. Stratifying lower UTIs by blood glucose levels, the results demonstrated that the higher the preoperative blood glucose level, the greater risk for acquiring postoperative lower UTIs; details are shown in Table 2.

### 3.2. Relationship among Frailty and Selected Factors for Lower UTIs

Firth’s logistic regression model was performed to ascertain the effect of preoperative frailty, DM, complications during hospital admission, and blood glucose levels on the likelihood that participants develop postoperative lower UTIs (Table 3). The model explained 33.9% (Nagelkerke R^2^) of the variance in postoperative UTIs and correctly classified 88.5% of cases. The unadjusted analysis revealed that complications during admission, a very high blood glucose level, and severe frailty were significantly associated with developing UTIs postoperatively (*p* < 0.05). Exploring variables in the model and adjusting for age, gender, and having DM, the findings demonstrated that the complication during admission and frailty significantly increased the risk of developing lower UTIs post-surgery (*p* < 0.05). Older surgical patients who developed complications during admission were at a 10.57-fold higher risk for developing postoperative lower UTIs (*p* < 0.001). Those with severe frailty preoperatively had a 3.46-fold greater risk (*p* < 0.05), and frailty was associated with a 1.14-fold risk (*p* = 0.817) for developing postoperative lower UTIs compared with being pre-frail. However, age, gender, having DM, a high blood glucose level and a very high blood glucose level were not significantly associated with the development of postoperative lower UTIs in older adults undergoing noncardiac surgery (*p* > 0.05).

## 4. Discussion

This study assessed the prevalence of lower UTIs and the association among age, gender, DM, complications during hospital admission, preoperative frailty status, and blood glucose level, with lower UTIs in older noncardiac surgical adults. The findings of the current study revealed that lower UTIs after a noncardiac surgery were common in this population. Moreover, severe frailty and the presence of any complication during hospital admission were significantly associated with lower UTIs.

The prevalence of lower UTIs postoperatively was approximately 15% in the current investigation. However, the prevalence of lower UTIs following noncardiac surgery may vary depending on the clinical setting, bladder catheterizations, or populations. Previous research reported that older adults undergoing colon cancer surgery frequently developed postoperative UTIs (approximately 17%), which were a primary cause of more severe complications, including respiratory infections and sepsis [11]. In Thailand, an infection epidemiology study of hospitalized older persons in tertiary care hospitals revealed that UTIs were the primary cause of in-hospital mortality (16.74%), with a prevalence of 43.9% [32]. In orthopedic surgery, the prevalence of postoperative UTIs was found in hip-fracture surgery to be 28.2% [33] and in spine surgery to be 8.2–11.8% [34,35], while the prevalence of UTIs was 11% in urogynecological surgery [36], and a 26% incidence rate was found in colorectal cancer surgery [37]. The disparity in UTI prevalence may be due to varying preoperative care protocols among hospitals and populations. Moreover, the complexity of complicated or asymptomatic UTIs in older adults may be problematic for a definite diagnosis [6], which may delay an effective response and appropriate interventions. The prevalence of lower UTIs consequently needs to be interpreted with caution. Further inquiry with a greater focus on investigating biological risk factors influencing lower UTIs or comparing the preoperative protocol and catheterization status of those patients is thus suggested.

The most prominent finding from the current analysis was that patients with a severely frail status prior to surgery who developed any other postoperative complication displayed an increased risk for lower UTIs post-surgery. These findings corroborated those of a large number of previous studies of postoperative complications in older adults across multiple surgeries, which highlighted that preoperative frailty significantly predicted postoperative complications of either minor or life-threatening events [13,16,17,18,38,39,40]. Clearly, the more severe the frailty, the higher the risk for the occurrence of postoperative complications. Moreover, such personal characteristics as increased age, malnutrition, or multiple comorbidities were more likely to enhance the risk of developing frailty and postoperative problems in older surgical patients [19]. These findings raise intriguing questions regarding the nature and extent of the complexity of aging and the nexus that underpins age-related decline and surgical outcomes in older adults undergoing noncardiac surgery. A further study with a greater focus on intervention or advanced surgical planning to modify preoperative risk factors for preventing postoperative complications in noncardiac surgery will need to be undertaken.

Considering the frailty status, the finding of the present study was in agreement with previous studies using mFI-11 in terms of the prevalence of frailty. Moreover, our findings also demonstrated that the more severe the frailty status of a person, the higher the risk of developing lower UTIs. These present findings broadly support the work of other studies in older surgical adults associating frailty and UTIs. The earlier study of Shahrestani et al. [35] investigated the prevalence of frailty and UTIs in older adults with lumbar spine surgery; the findings showed that 11.8% of this population was frail with UTIs. Additionally, frail older adults were 3.97 times more likely to develop UTIs after surgery than non-frail older adults (OR: 3.97, 95% confidence interval: 3.21–4.95, *p* < 0.0001). Frailty was also associated with an increased risk of developing hospital-acquired catheter-associated UTIs within 2 weeks of admission and a 1.40 times higher risk for in-hospital mortality in hospitalized older adults [41]. Other prior studies have found that such complications coexisted with frailty rather than UTIs. A number of studies revealed that preoperative frailty, which was assessed by mFI-11, was also associated with such adverse outcomes as UTIs, respiratory complications, and surgical site infections [11,13,35,42]. Recent evidence revealed that older adults with DM and displaying preoperative frailty are at risk for developing UTIs and increased hospital stays [17,18,38,40]; moreover, the greater the severity of frailty, the higher the risks for mortality, a 30-day ICU admission, and health care utilization [17,18,38]. Our findings have an important implication for implementing a preoperative frailty assessment to prevent postoperative complications, not only UTIs but also others for noncardiac surgery in the older population. Importantly, the preoperative frailty prevalence must be treated with caution because the concept of frailty applied in the present study involved multiple diseases in classifying the severity of frailty. Therefore, the prevalence of preoperative frailty and its severity may differ from other studies that applied different concepts and instruments to measure frailty; the notion of our study was congruent with a scoping review study of frailty-measuring instruments used in orthopedic surgery [43]. In order to acquire a more comprehensive understanding of preoperative frailty, additional research is required to compare the various approaches to frailty assessment in this population.

Regarding the relationship between the levels of frailty and lower UTIs, when controlling for other variables, the findings of the present study demonstrated this relationship to be similar to several earlier studies, indicating that the preoperative frailty status is significantly associated with an increased risk for lower UTIs [16,17,18,33,36,37,40,41,44]. These notions further support the idea that frailty is an age-related decline, which occurs across multiple bodily systems, resulting in an increased risk of developing complications, dependency, disability, and poor quality of life [11,14,39,45]. Participants with frailty are vulnerable to many stressors; thus, exposure to external or internal stressors—hospital admission, surgical procedures, and imbalanced homeostasis—is more likely to increase adverse outcomes [23,24]. Moreover, a South Korean study revealed that frailty frequently coexists with hypertension, osteoporosis, or diabetes, which increases the risk for adverse outcomes [46]. Therefore, older surgical adults who present with preoperative frailty are more vulnerable to postoperative complications such as UTIs, particularly those with co-morbid diseases such as DM. Additionally, other known factors include the presence of multiple comorbidities, chronic opioid use, bleeding conditions, hemolytic conditions, iron deficiency, and B12 deficiency, altered plasma glucose, and glycated hemoglobin in older adults [17]. Notably, frailty-associated UTIs are prevalent in frail older persons, making them difficult to manage effectively. Older adults with severe frailty and those at risk of having a long-term catheter, dehydration, incontinence, or bladder outflow obstruction are more prone to developing complicated UTIs and sepsis [47]. Hence, it would be prudent to identify frailty early in order to avoid preventable complications, such as UTIs, in this population. Recent evidence has highlighted that integrating the notions of frailty and the level of frailty is essential in enhancing appropriate interventions, medical therapeutics, and glycemic management for older adults with DM [17].

Despite the favorable findings, our study discovered that managing glucose levels in older surgical adults with DM who presented with a frail status appears more challenging. In the present study, the findings revealed that most of the participants had normal blood glucose levels even if they displayed frailty. Although either high or very high blood glucose levels were highly likely to be associated with the development of postoperative lower UTIs [48], the present study did not find an association between high blood glucose levels and an increased risk for lower UTIs. Additionally, frail older adults demonstrated varying blood glucose levels, but not all participants with frailty developed lower UTIs. These findings contradict recent studies that suggested that blood glucose level alterations among frail older adults with DM are more likely to increase the risk for infections [17,18,38,40,41]. A possible explanation for these findings could be that the surgical stress response plays a significant role not only in influencing the alteration of the inflammatory–immune system, resulting in an increased risk for infection or excessive inflammation, but also in enhancing the neuroendocrine–metabolic response, resulting in increased blood glucose and diminished energy resources [25]. Moreover, one reason why increased age, sex, and having DM were not significantly associated with lower UTIs post-surgery is that frailty may be a substantial element in increasing dysregulated homeostasis across bodily systems. Therefore, the more severe the frailty, the more likely the risk for infection and increased blood glucose after receiving surgery [25,49]. Therefore, monitoring and controlling glucose levels have become increasingly critical, particularly in geriatric surgery with frailty. Current evidence suggests that frailty and DM share potential mechanism factors—low-graded chronic inflammation and impaired oxidative stress resistance [49]. Frailty and DM appear to be the two sides of the coin; thus, an inflammation biomarker investigation is also required. Moreover, applying standard preoperative care in high-level tertiary care hospitals may prevent uncontrolled blood glucose levels, resulting in diminished risks of developing lower UTIs. Because the level of frailty was significantly associated with postoperative UTIs, the early identification of frailty at the preoperative stage will improve care quality and prevent common postoperative complications such as lower UTIs in this population.

## 5. Conclusions

The present study provided new insights into the factors associated with lower UTIs in a tertiary care hospital as well as enhancing the understanding of the complexity of aging and surgical outcomes. In summary, this study revealed that severe preoperative frailty and having any postoperative complications significantly predicted lower UTIs in older adults undergoing major noncardiac surgery when controlled for age, gender, and diabetes mellitus. Because the consequences of in-hospital UTIs may be far beyond expectation regarding profound health problems in older adults, proactive professional nursing care is required. Effective preoperative intervention that combines the notion of frailty may be more beneficial in avoiding preventable postoperative complications such as lower UTIs in this population. Moreover, the findings of the present study may shed light on strengthening professional nursing roles—gerontological nurse practitioners or advanced practice nurses—to prevent postoperative lower UTIs by implementing a practical frailty assessment into daily clinical practice to enhance better preoperative quality care, prevent postoperative complications, and promote quality of life. Notably, these findings have significant implications suggesting that developing a preoperative intervention that integrates frailty-specific glycemic management may be essential to strengthening patient-centered care in this population.

## Figures and Tables

**Table 1:** Characteristics of study participants (n = 220).

**Characteristics**		**Characteristics**	
**Gender**		**Marital status**	
Male	83 (37.7)	Single	15 (6.8)
Female	137 (62.3)	Married	160 (72.7)
**Age (years)**		Divorced/Separated	42 (20.5)
60–69	76 (34.5)	**Education**	
70–79	99 (45.0)	Primary school	144 (65.5)
>80	45 (20.5)	Highschool	39 (17.7)
(Range 60–94, Mean = 72.95; SD = 7.253)		Bachelor’s degree	35 (15.9)
**Number of comorbidities**		>Bachelor’s degree	2 (0.9)
1 disease	62 (28.2)	**Type of surgery**	
2 diseases	80 (36.4)	Gastroenterology	90 (40.9)
3 diseases or more	78 (35.4)	Orthopedic	64 (29.1)
BMI (kg/m^2^) *		Breast and Endocrine	16 (7.3)
<18.5 (Underweight)	15 (6.8)	Neurology	12 (5.5)
18.5–22.9 (Normal)	71 (32.3)	Thoracology	8 (3.6)
23.0–24.9 (Overweight)	45 (20.5)	Otolaryngology	18 (8.2)
25.0–29.9 (Obese I)	65 (29.5)	Urology	6 (2.7)
≥30.0 (Obese II)	24 (10.9)	Vascular	6 (2.7)
(Median = 23.95, Mean = 24.45; SD = 4.55)		**Post-operative complication**	
**Activities of daily living**		Yes	46 (20.9)
Independently	198 (90)	No	174 (79.1)
Partial dependently	21 (9.5)	Types of complications during admission **	
Severe dependently	1 (0.5)	Pressure injury	4 (8.69)
**Length of Hospital Stay (day)**		Infection	7 (15.2)
<7	102 (46.4)	Re-operation	4 (8.69)
7–15	70 (31.8)	Respiratory failure	12 (26.1)
>15	48 (21.8)	Delirium	4 (8.69)
(Median = 7, Mean = 11.9; SD = 14.7)		VAP/HAP	3 (6.52)
		Arrythmia	4 (8.69)
		Other complication	12 (26.1)
**Blood glucose group (mg%)**		**Pre-operative urinary tract infection**	
Normal (70–126)	157 (71.4)	Yes	14 (6.4)
High (127–200)	54 (24.5)	No	206 (93.6)
Very high (>200)	9 (4.1)	**Post-operative urinary tract infection**	
**Frailty level**		Yes	33 (15.0)
Pre-frail	102 (46.4)	No	187 (85.0)
Frail	81 (36.8)		
Severely frail	37 (16.8)		

* BMI = Body Mass Index with ASIA criteria classification (WHO, 2004); ** One participant may have one or more postoperative complications.

**Table 2:** Urinary tract infections in older adults undergoing major noncardiac surgery stratified by frailty and blood glucose levels.

Variables	No Infection (n = 187) Number (%)	Lower-Urinary Tract Infection (n = 33) Number (%)
**Frailty level**		
Pre-frail	92 (49.20)	10 (30.30)
Frail	71 (37.97)	10 (30.30)
Severely frail	24 (12.83)	13 (39.40)
**Blood glucose level**		
Normal (70–126 mg %)	137 (73.26)	20 (60.60)
High (127–200 mg %)	45 (24.07)	9 (27.28)
Very high (>200 mg %)	5 (2.67)	4 (12.12)

**Table 3:** Firth’s logistic regression analysis of factors influencing postoperative lower urinary tract infections in older adults undergoing noncardiac surgery, after controlling for other variables (N = 220).

Variables	Univariate ORs (95%CI)	*p*-Value	Adjusted ORs (95%CI)	*p*-Value
Age	0.98 (0.92–1.03)	0.978	0.97 (0.91–1.02)	0.256
Female gender	0.93 (0.43–2.02)	0.789	1.03 (0.43–2.49)	0.947
Diabetic Mellitus (DM)	1.22 (0.56–2.59)	0.608	0.80 (0.26–2.39)	0.717
Complications during admission	13.58 (5.99–32.56)	<0.001	9.53 (4.06–23.28)	<0.001
Blood glucose level				
Normal	Reference			
High (120–200 mg %)	1.37 (0.56–3.15)	0.470	0.91 (0.30–2.51)	0.862
Very high (>200 mg %)	5.48 (1.27–22.44)	0.017	1.71 (0.29–10.34)	0.541
Frailty				
Pre-frail	Reference			
Frail	1.29 (0.51–3.32)	0.584	1.14 (0.35–3.60)	0.817
Severely frail	4.98 (1.96–13.05)	<0.001	3.46 (1.03–11.95)	0.045

R analysis adjusted variables: Model fitted by Penalized ML; 1-Wald, 2-Profile penalized log-likelihood: Likelihood ratio test = 46.11337 on 7 df, *p* < 0.001; Wald test = 78.13931 on 7 df, *p* < 0.001. Adjusted variables: age, gender, and diabetic diseases. Abbreviations: ORs = Odds ratios; CI = Confidence Interval.

## Data Availability

Data are available upon request due to restrictions, e.g., privacy or ethical concerns.
